# Computational Process of Sharing Emotion: An Authentic Information Perspective

**DOI:** 10.3389/fpsyg.2022.849499

**Published:** 2022-05-12

**Authors:** Shushi Namba, Wataru Sato, Koyo Nakamura, Katsumi Watanabe

**Affiliations:** ^1^Psychological Process Research Team, Guardian Robot Project, RIKEN, Kyoto, Japan; ^2^Department of Cognition, Emotion, and Methods in Psychology, Faculty of Psychology, University of Vienna, Vienna, Austria; ^3^Japan Society for the Promotion of Science, Tokyo, Japan; ^4^Faculty of Science and Engineering, Waseda University, Tokyo, Japan; ^5^Faculty of Arts, Design and Architecture, University of New South Wales, Sydney, NSW, Australia

**Keywords:** sharing emotion, facial expressions, authenticity, MPT model, emotion

## Abstract

Although results of many psychology studies have shown that sharing emotion achieves dyadic interaction, no report has explained a study of the transmission of authentic information from emotional expressions that can strengthen perceivers. For this study, we used computational modeling, which is a multinomial processing tree, for formal quantification of the process of sharing emotion that emphasizes the perception of authentic information for expressers’ feeling states from facial expressions. Results indicated that the ability to perceive authentic information of feeling states from a happy expression has a higher probability than the probability of judging authentic information from anger expressions. Next, happy facial expressions can activate both emotional elicitation and sharing emotion in perceivers, where emotional elicitation alone is working rather than sharing emotion for angry facial expressions. Third, parameters to detect anger experiences were found to be correlated positively with those of happiness. No robust correlation was found between the parameters extracted from this experiment task and questionnaire-measured emotional contagion, empathy, and social anxiety. Results of this study revealed the possibility that a new computational approach contributes to description of emotion sharing processes.

## Introduction

When encountering someone who (apparently) smiles, perceivers often replicate the smile on their own face and thereby feel happiness. This phenomenon is known as emotional contagion (Hatfield et al., 1993). Emotional contagion has long been regarded as reflecting a mimicry-based process, for which mimicry of emotional expressions and its consequent feedback function are assumed (Hatfield et al., 1993, 2014). However, emotional contagion can be evoked by higher-order social processes (Deng and Hu, 2018) or by a simple emotion-to-action response as well as the primary mimicry-based process (Dezecache et al., 2016; Isern-Mas and Gomila, 2019). Consequently, emotional contagion is assumed to occur through multiple processes by the sharing of emotional states between two persons (or more; Dezecache et al., 2013; Coviello et al., 2014).

Research investigating phenomena of emotional contagion have mainly targeted a single modality such as facial expressions (e.g., Hess and Blairy, 2001; Deng and Hu, 2018) in isolation. Yet, emotional contagion occurs by multiple modalities including voice (Rueff-Lopes et al., 2015) and text information (Cheshin et al., 2011). In fact, Kramer et al. (2014) presented experimental evidence for large-scale emotional contagion *via* social networks, where emotional contagion can enhance the social glue connecting users through emotional state sharing.

Earlier studies of emotional contagion have often argued the underlying mechanisms without consideration of whether a target person and perceivers actually share an emotional experience. According to Theory of Affective Pragmatics, people generally extract diverse affective information from other persons’ facial expressions (Scarantino, 2017, 2019): what facial expressions convey to a perceiver strongly influences affective communications (Jack et al., 2014; Hess, 2021). Emotional expressions that have been studied in emotional contagion studies are often spontaneous expressions (e.g., Hess and Blairy, 2001), but they have not verified whether perceivers can detect the existence of actual feeling states from these expressions (Lishner et al., 2008; Dezecache et al., 2013; Deng and Hu, 2018). In fact, several studies have demonstrated clearly that the inference that “the emotion is expressed” should be different from the inference that “the person who expresses the emotion is actually experienced” (McLellan et al., 2010; Namba et al., 2018). In principle, if emotional contagion (or emotional convergence) has been assumed to be a consistent emotional state between perceivers and a target person, then the perception of authentic information from targets’ facial expressions as perceived-as-genuine expressions can be expected to be a prerequisite for sharing the same emotional state. Therefore, to investigate the cognitive processes underlying sharing of emotion, the transmission of authentic information from emotional expressions must be considered.

Earlier studies found that facial expressions which are perceived as genuine can have stronger effects on the perceivers’ psychological reactions than non-genuine ones. For instance, Miles (2009) reported that static enjoyable smiles which included cheek raising are likely to be regarded as more approachable than static non-enjoyment smiles which did not include cheek raising. In addition, Krumhuber et al. (2009) demonstrated that temporal features of authentic smiles, rather than fake smiles, elicited positive attributions and benefits for job interview situations. Static genuine tearful expressions, more than insincere displays, can elicit helping and empathic responses from perceivers (Krivan and Thomas, 2020). Regarding other sources of emotional information such as vocalization, McGettigan et al. (2015) found distinct neural responses to genuine and deliberate laughs. Additionally, Lima et al. (2021) revealed that authentic laughing induces stronger facial and skin conductance responses than posed laughing. Regarding emotional contagion, Namba and Kabir (2019) reported that dynamic facial displays of genuine happiness and surprise rather than their posed counterparts were found to have a significant positive correlation between facial mimicry and self-reported feeling states. This finding indicated that authentic expression elicited relations between facial mimicry and emotional contagion, which supported the interpretation of mimicry-based contagion processes. Given evidence related to perceived-as-authentic expressions, the judgment of authenticity in emotional expressions can be crucially important to the process of sharing emotional states.

The Multinomial Processing Tree (MPT) model is a powerful framework used to describe the emotional contagion process. The MPT model can stipulate how underlying component processes interact to shape behavioral outcomes on a task and can estimate latent variables suited for psychological interpretation (Batchelder and Riefer, 1999). This commonly used model has been applied to study cognitive processing for tasks such as source monitoring (Bröder and Meiser, 2007; Erdfelder et al., 2009), but it has also been used in the social cognition area. For example, the MPT model can predict empathy for pain (Cameron et al., 2017), emotion recognition (Matsunaga et al., 2018), and trustworthiness impression formation (Klapper et al., 2016). Given that the MPT is a widely applicable model, MPT model will also be well-fitted for sharing emotional states.

Although similar feeling states often occur between perceivers and a target person through dynamic facial expressions (e.g., Rymarczyk et al., 2019), underlying processes by which the experience is generated remain unclear. Two (or more) possible processes can take place in perceivers when facial expressions serve as emotion-elicitation stimuli. The one process is the forward process, which elicits directly similar feeling states irrespective of the perception of authentic information about expressers’ feeling states. Another process is the empathetic process, which shares the experience relying on the perception of authentic information for expressers’ feeling states (Elfenbein, 2014). Compared to a simple model such as linear regressions, the MPT model has been a powerful tool for describing such multi-stage processes that result in similar observations. Application of this model enables us to dissociate the sharing process of feeling states from the simple emotion elicitation (not-sharing) process and enables us to quantify the occurrence of each process.

**Figure 1:**
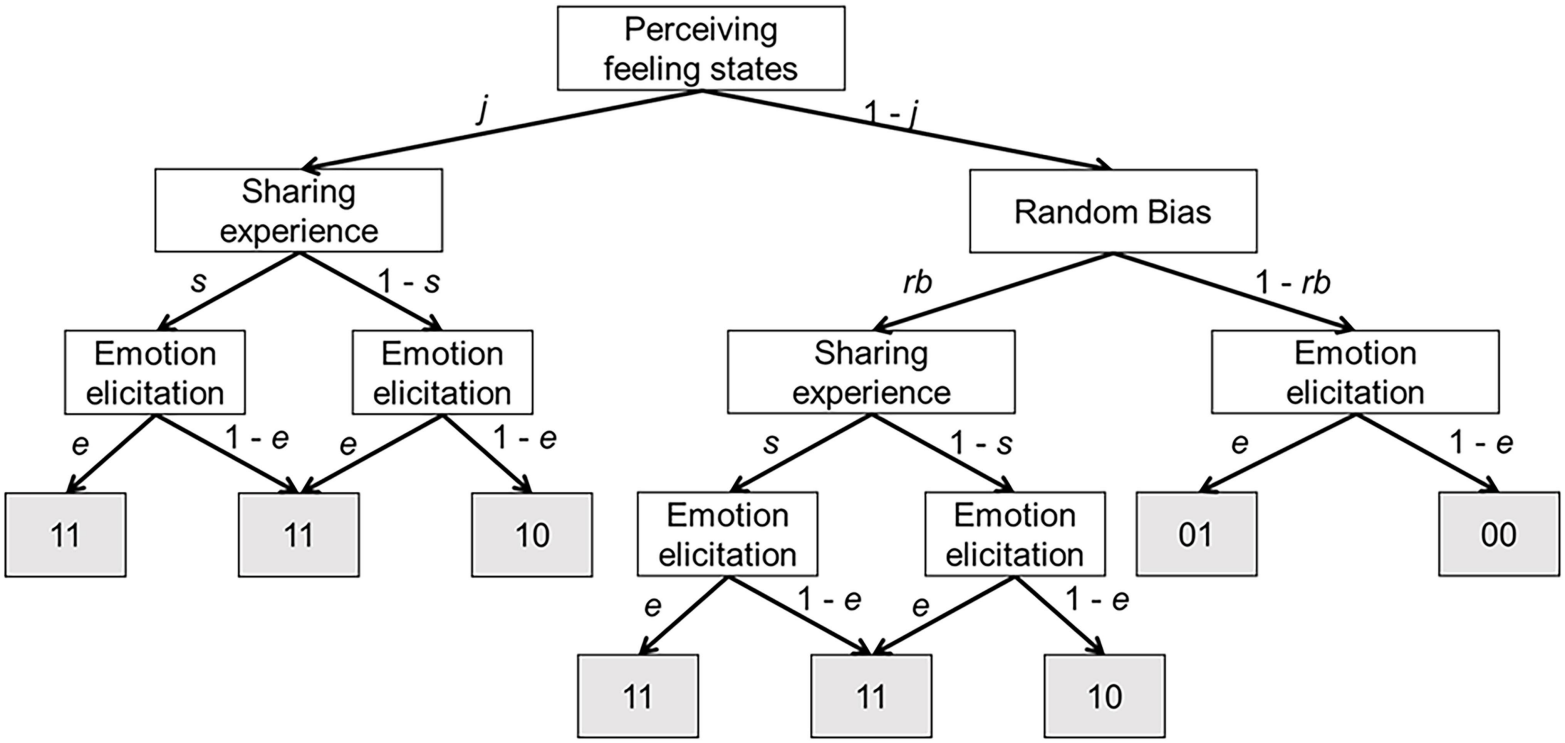
Schematic representation of the model used for this study. White squares represent the latent states; and gray squares represent the observed variables. The first number of gray colored observation means “detecting an emotional experience from perceived emotional facial expressions (1) or not (0),” whereas the second number means “the same emotional experience for the emotional expression occurred in the perceiver (1) or not (0).” Consequently, the gray square containing “01” represents the following observed variable: “the experience was not detected from the emotional expression (0), but the same emotional experience occurred (1).” In a hierarchical model, each participant has its own parameters.

This study was conducted to elucidate the underlying processes of sharing emotional states by application of the model, which emphasizes the perception of authentic information for expressers’ feeling states from facial expressions. For that purpose, we expected that the MPT model would be able to decompose behavioral outcomes to underlying processes. Figure 1 presents an illustration of the process that we examined specifically in this study. Table 1 presents a list of psychological interpretations of all parameters. The assumptions are following: (1) before sharing emotional states, perceivers must decide whether a target person has a specific feeling state; (2) perceivers show a response bias in perceiving some feeling states to a greater or lesser degree, even from a neutral expression that drives slight or no facial movements (e.g., Said et al., 2009; Albohn et al., 2019; Hester, 2019); and (3) if perceivers can detect authentic information about feeling states from a target person and thereby feel similar valence experiences, such congruent responses can be regarded as sharing emotional states. In contrast, if perceivers are unable to detect authentic information about feeling states from a target person but feel the emotion as the target’s expression indicates, then it can be regarded as simple emotion elicitation by emotional messages (Dezecache et al., 2016).

**Table 1:** Psychological interpretation of the model parameters.

Parameter	Description
*j*	Probability of perceiving feeling states from an emotional expression
*rb*	Probability of perceiving feeling states from no facial movement (a neutral expression)
*s*	Probability of sharing feeling states from an emotional expression
*e*	Probability of eliciting feeling states from an emotional expression

Using the MPT model, the current study tests five hypotheses. First, the process to perceive authentic information about feeling states is expected to be differentiated between anger and happy expressions. Calvo et al. (2013) showed distinct event-related potentials associated with the perception to static genuine and ambiguous smiles. Results of another study indicated that the neural processing encoding positive and negative valence expressions differs (Schupp et al., 2004). In fact, Dawel et al. (2017), who applied McLellan’s dataset, demonstrated that the authenticity judgment differs between happiness and anger using static facial images. Perceivers are able to discriminate event-elicited and posed happy expressions, although they cannot decipher the authentic information from angry expressions. Given the evidence presented above, one can reasonably estimate the parameters for detecting authentic information separately from angry and happy expressions in the process of sharing emotional states. Consequently, hypothesis 1 is the following.

1. Angry and happy expressions have distinct inferential processes for perceiving authentic information about feeling states.
  Murphy et al. (2018) pointed out that the types and functions of an emotional contagion phenomenon are dependent on emotional valence. Deng and Hu (2018) also supported the existence of two distinct processes of emotional contagion, depending on positive and negative emotional processing. Indeed, earlier studies addressed facial mimicry as an important process of emotional contagion (Hatfield et al., 1993), where people are more likely to mimic happy expressions than angry expressions (Seibt et al., 2015). Hypothesis 2 is the following.
2. Processes of sharing and eliciting emotional states differ for anger and happiness.
  In general, Bayesian hierarchical modeling provides individual differences and similarities among participants and leads to more accurate statistical inferences (Gelman and Hill, 2007; Driver and Voelkle, 2018). Moreover, a full hierarchical model with multivariate priors can provide a correlated pattern between estimated parameters, thereby yielding analysis results that are well-suited to real world data (Gelman and Hill, 2007). In fact, Laird et al. (1994) identified non-negligible individual variations in emotional contagion processes. Therefore, we tested the following two hypotheses as well.
3. Data have a hierarchical structure allowing variation among individual perceivers.
4. Data have a correlated structure for each estimated parameter fitting multivariate priors.
  Finally, some personality traits related to social cognition are expected to modulate parameters involved in the process of sharing emotional states or simple elicited emotion. Persons with high trait-empathy are known to be more likely to mimic emotional expressions than their low trait-empathy counterparts (Dimberg et al., 2011; Rymarczyk et al., 2016). Manera et al. (2013) reported that the susceptibility to emotional contagion for negative emotions (Doherty, 1997) improves smile authenticity detection. Neves et al. (2018) also described that people who score highly on both the emotional contagion scale and empathic concern scale of the Interpersonal Reactivity Index (Davis, 1980, 1983) show higher ability to discriminate the authenticity of laughs. Dawel et al. (2019) reported that genuine smilers were judged as more friendly than posed ones. The tendency was positively correlated with the score of social anxiety. Therefore, several questionnaire items related to emotional contagion (Doherty, 1997), empathy (Davis, 1980, 1983), and social anxiety (Mattick and Clarke, 1998) can be expected to modulate each parameter of sharing emotional states that is estimated using the MPT model.
5. Patterns of correlation exist between parameters computed using the MPT model and the personality traits.
  To test the five hypotheses presented above, this study compared the models that incorporated perceivers’ perception of authentic information and feeling states from anger and happy facial expressions. Furthermore, we investigated the relation between the estimated parameters by the MPT model and several personality traits. We predicted that all hypotheses would be verified by building and comparing computational models. Regarding Hypothesis 5, traits related to emotional contagion and empathy were expected to be positively related with all parameters inherent with sharing of emotion, whereas social anxiety could heighten the ability to detect feeling states. We anticipated that these patterns would be consistent with earlier findings (Manera et al., 2013; Calvo et al., 2018; Neves et al., 2018; Dawel et al., 2019).

## Materials and Methods

### Participants

We collected data from 89 crowdsourcing workers (64 women and 25 men, age 19–73 years, Mean = 37.92, SD = 10.79) who consented to participate in a survey *via* Crowdworks (CW).^1^ One female participant was excluded because of experimental error. All participants were Japanese. Regarding the crowdsourcing sample quality, Majima et al. (2017) confirmed earlier that CW participants are aligned with normal Japanese participants in in-lab behavioral experiments. Informed consent to the study procedures on the CW platform was obtained from each participant before the investigation, in line with a protocol approved by the Ethical Committee of the Graduate School of Education, Hiroshima University (2019086), and the Institutional Review Board of Waseda University (2015–033). This study was conducted in accordance with the ethical guidelines of our institute and the Declaration of Helsinki. After completing the experimental task, participants received 900 JPY for completing a 60-min survey. Because the current study was the first attempt to describe sharing emotion using the MPT model, it was difficult to estimate the effect size. Therefore, the following power analysis was performed to calculate the sample size to check hypothesis 5 (correlation analysis). Power analysis using software (G*Power; Faul et al., 2007) demonstrated that our sample size was sufficient to detect correlation of 0.3, with 80% power for two-tailed tests at *p* = 0.05, resulting in required *N* of 84.

### Stimuli

For this study, we used recorded video clips of facial expressions made by 16 Japanese models (50% women: age = 21–33 years, mean = 26.60, SD = 3.22) who were well-trained as semi-professional actors. All models were instructed to express facial expressions according to the short descriptions inherent to six emotions (anger, happiness, disgust, fear, sadness, and surprise). Experimenters prepared stories of four kinds for each emotion and recorded each facial expression of emotion, and also recorded neutral facial expressions that included no facial movements four times. It is noteworthy that all these expressions have been regarded as posed expressions. The recording settings were 1,920 × 1,440 pixel resolutions at 30 frames per second. Target video sequences were 4-s videos with a peak frame in the middle. Because of time constraints on the viewed clips, this study used emotions of only three types: anger, happiness, and neutral. The main experiment used 16 (models) × 2 (emotion: anger, happiness) × 4 (scenarios) plus neutral expressions by 20 (models) which added further four models (50% women): a total of 148 total clips. Angry scenarios were the following: “when you are blamed even though you are not at fault at all,” “when someone insults your family,” “when you find out that someone has been deceiving you all along,” and “when things did not go your way and you finally did not achieve your goal.” Happy scenarios were the following: “when you enjoy conversation with your friends,” “when someone praises you,” “when you win a match,” and “when you see a friend you have not seen for years.” For checking the validation of the stimuli, we compared each prototypical facial component (Ekman et al., 2002) at the apex frame with the neutral expressions using automated facial action detection systems (Baltrusaitis et al., 2018; Namba et al., 2021). The results demonstrated that happy and angry displays were significantly likely to include prototypical facial actions (anger: frowning, lid tightening, and raising upper lip, *t*s > 2.83, *p*s < 0.006; happiness: cheek raising and lip corner pulling, *t*s > 5.68, *p*s < 0.001).

### Procedure

**Figure 2:**
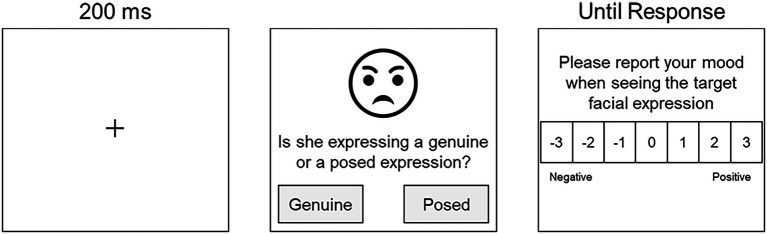
Schematic diagram of a typical trial.

The experimental program was created using software (Gorilla Experiment Builder^2^; Anwyl-Irvine et al., 2020). Before starting experiment trials, all participants read the information sheet on the first page of the survey on Gorilla. They were asked to give informed consent for their participation *via* a check-box. This form of consent was approved by the Ethical Committee of the Graduate School of Education, Hiroshima University (2019086). On the platform, the participants were asked to provide basic information about themselves (age and sex). Next, they were given careful explanations about the concept of genuine and posed facial expressions and their requirements as participants, according to the instructions used by Namba et al. (2018). The following explanation was given in Japanese: “This study aims to find out what feeling states are caused by seeing facial expressions. People sometimes express genuine facial expressions triggered by actual emotional experiences, although people might express posed facial expressions of emotion by intentional manipulation in other situations. … In this study, we also aim at understanding whether people have the ability to detect a person’s emotional feeling.” Participants were blind to how much genuine or posed facial expressions were included. We asked participants to perform tasks of two types related to the perception of facial expressions (Figure 2). The first task was to judge whether the target persons were showing either genuine or posed expressions (e.g., “Is she expressing genuine or posed expression?”). The second task was to estimate the intensity of feeling states the participant feels from the facial expression in terms of valence (e.g., “Please answer your mood when you see the target facial expression”) on a scale from −3 (very negative) to 3 (very positive). Self-reported pleasant and unpleasant moods were used as an index of emotional contagion (Hennig-Thurau et al., 2006; Rueff-Lopes et al., 2015; Lischetzke et al., 2020). The current study applied the seven-point scale because a positive relation exists between the number of scale points and the measurement reliability (Churchill and Peter, 1984). A reasonable number of categories can be seven (Preston and Colman, 2000). The use of a scale with more than seven points can be less meaningful to raters (Krosnick and Presser, 2010). To familiarize participants with the task flow, they performed practice trials with two facial stimuli that were never used in the main trials (two intended smiles expressed by the experimenter). The main task program started if the participants fully understood the task. In the main experiment, participants were presented with one facial expression from a pool of 148 dynamic facial stimuli. Then the participants were required to perform the two tasks. The order of facial stimuli was randomized. All clips were played once. They disappeared when the participants made their decisions. Participants were unable to change their answers after they had input a response. The inter-stimulus interval was about 200 ms. After completing the main experiment, the participants were asked to complete the following four questionnaires to assess the individual trait differences related to social cognition: the Emotional Contagion Scale (ECS; Doherty, 1997) for sensitivity to others emotional states, the Interpersonal Reactivity Index (IRI; Davis, 1980, 1983) for empathy, and the Social Interaction Anxiety Scale and the Social Phobia Scale (SIAS and SPS: Mattick and Clarke, 1998) for social anxiety.

This study applied the Japanese translated versions of the ECS (Kimura et al., 2007), IRI (Himichi et al., 2017), and SIAS and SPS (Kanai et al., 2004). The first, ECS, has been developed and validated as the 15-item unidimensional measure of susceptibility to others’ emotion (Doherty, 1997). IRI is a 28-item measure of four empathy dimensions: Empathic Concern, Perspective Taking, Personal Distress, and Fantasy (Davis, 1980, 1983). Mattick and Clarke (1998) developed the 20-item Social Phobia Scale and the 20-item Social Interaction Anxiety Scale to assess social anxiety.

Table 2 presents descriptive statistics for all questionnaires as average scores of the items. As shown, it is noteworthy that the perspective taking scale was not highly reliable, which is consistent with results reported from an earlier study (Himichi et al., 2017). The Supplementary Material provides comparisons with earlier studies and a correlation matrix of these scales for checking the characteristics of these scales. In keeping with open science practices that emphasize the transparency and replicability of results, all data have been made available online.^3^

**Table 2:** Internal consistency (Cronbach’s Alpha) and descriptive statistics for questionnaire responses.

Subscale	No. of items	Cronbach’s *α*	Mean	SD
ECS	15	0.85	2.63	0.52
Empathic concern (IRI)	7	0.77	3.46	0.65
Perspective taking (IRI)	7	0.66	3.06	0.62
Personal distress (IRI)	7	0.77	3.20	0.75
Fantasy (IRI)	7	0.78	3.19	0.79
SPS	20	0.90	1.03	0.60
SIAS	20	0.93	1.98	0.81

### Statistical Approach

To build the MPT model, we adopted a Bayesian approach to evaluate uncertainty as probability distributions. By navigating through the branches of the proposed model (Figure 1), the categorical outcomes are described as presented below:

*00 = Perceiver did not detect feeling states from a target expression and did not have the same emotional states.*

*01 = Perceiver did not detect feeling states from a target expression but had the same emotional states.*

*10 = Perceiver detected feeling states from a target expression but did not have the same emotional states.*

*11 = Perceiver detected feeling states from a target expression and had the same emotional states.*

The objective criterion by which the corresponding emotional states were elicited was defined as whether the perceivers responded similar feeling states to valence of the target expression. For example, in the case of happy expressions, the score was more than 1 for a reported emotional state as “elicited the same emotional states”; in the case of angry expressions, the score was less than −1 for a reported emotional state as “elicited the same emotional states.” Using these outcomes, the probabilities of the four responses were calculated using the underlying parameters presented in Table 1.


P(00|j,rb,s,e)=(1−j)∗(1−rb)∗(1−e)

P(01|j,rb,s,e)=(1−j)∗(1−rb)∗e

P(10|j,rb,s,e)=(1−j)∗rb∗(1−s)∗(1−e)+j∗(1−s)∗(1−e)

$$\begin{array}{c} P\left(11|j,rb,s,e\right)=j*s*e+j*s*\left(1-e\right)+j*\left(1-s\right)*e+\left(1-j\right)*rb*s*e\\ +\left(1-j\right)*rb*\left(1-s\right)*e+\left(1-j\right)*rb*s*\left(1-e\right). \end{array}$$

These probabilities sum up to 1. However, we were interested only in the response bias to perceive the authentic information of some feeling states from neutral facial expressions. Regarding only trials for neutral expressions, we calculated the following probabilities.


P(Yes|rb)=rb

P(No|rb)=(1−rb).

In this model, we were unable to prespecify any prior of parameters. A Beta distribution with *α* = 2 and *β* = 2 was assumed for the parameters *j*, *rb*, *s*, *e*. This setting followed practices used for an earlier study (Nicenboim et al., 2021), and our sensitivity analysis in terms of WAIC confirmed this Beta distribution outperformed uniform distribution (i.e., *α* = 1 and *β* = 1). The proposed basic models (Model 1) were the following:


$$\theta_{1}=rb$$

$$\theta_{0}=\left(1-rb\right)$$

$$\theta_{00}=\left(1-j\right)*\left(1-rb\right)*\left(1-e\right)$$

$$\theta_{01}=\left(1-j\right)*\left(1-rb\right)*e$$

$$\theta_{10}=\left(1-j\right)*rb*\left(1-s\right)*\left(1-e\right)+j*\left(1-s\right)*\left(1-e\right)$$

$$\begin{array}{c} \theta_{11}=j*s*e+j*s*\left(1-e\right)+j*\left(1-s\right)*e\\ +\left(1-j\right)*rb*s*e+\left(1-j\right)*rb*\left(1-s\right)*e\\ +\left(1-j\right)*rb*s*\left(1-e\right) \end{array}$$

$$\theta_{\mathrm{Netural}}=\left\{\theta_{0},\;\theta_{1}\right\}$$

$$\theta_{\mathrm{Anger}}=\left\{\theta_{00\mathrm{Anger}},\;\theta_{01\mathrm{Anger}},\;\theta_{10\mathrm{Anger}},\;\theta_{11\mathrm{Anger}}\right\}$$

$$\theta_{\mathrm{Happiness}}=\left\{\theta_{00\mathrm{Happiness}},\;\theta_{01\mathrm{Happiness}},\;\theta_{10\mathrm{Happiness}},\;\theta_{11\mathrm{Happiness}}\right\}$$

y~Categorical(θ)

$$j,rb,s,e\sim \mathrm{Beta}\left(2,\;2\right).$$

To test hypothesis 1 (Angry and happy facial expressions have distinct inferential processes for perceiving authentic information about feeling states.), we added other assumptions that all parameters would differ depending on the valence type. Model 2 can be presented as shown below:


$$j_{\mathrm{anger}},j_{\mathrm{happiness}},rb,s,e,\sim \mathrm{Beta}\left(2,\;2\right).$$

In addition, to evaluate hypothesis 2 (Processes of sharing and eliciting emotional states differ for anger and happiness.), we constructed Model 3 by extending the assumption of Model 2.


$$\begin{array}{l} j_{\mathrm{anger}},rb_{\mathrm{anger}},s_{\mathrm{anger}},e_{\mathrm{anger}},j_{\mathrm{happiness}},\\ rb_{\mathrm{happiness}},s_{\mathrm{happiness}},e_{\mathrm{happiness}}\sim \mathrm{Beta}\left(2,\;2\right). \end{array}$$

Furthermore, we added a hierarchical structure to the underlying parameters in Model 3. The following model structure allowed for estimation of the differences between *n* participants, resulting in Model 4. We omitted differences of emotion (happiness and anger) for clarity.


$$\;\alpha_{j},\alpha_{rb},\alpha_{s},\alpha_{e}\sim \mathrm{Normal}\left(0,\;2\right)$$

$$\tau_{j},\tau_{rb},\tau_{s},\tau_{e}\sim \mathrm{Normal}\left(0,\;1\right)$$

$$u_{j,rb,s,e}\sim \mathrm{Normal}\left(0,\;\tau_{j,rb,s,e}\right)$$

$$j_{n},rb_{n},s_{n},e_{n}\sim {\mathrm{logit}}^{-1}\left(\alpha_{j,rb,s,e}+u_{j,rb,s,e;subj\left[n\right]}\right).$$

For Model 5, we extended Model 4 by inclusion of correlation among parameters (Gelman and Hill, 2007). The updating information for Model 5 was the information presented below.


$$\left(\alpha_{j},\;\alpha_{rb},\;\alpha_{s},\;\alpha_{e}\right)\sim \mathrm{MultiVariateNormal}\left(0,\;\Sigma \right)$$

LKJCorr(Σ|2).

All iterations were set to 5,000 and burn-in samples to 5,000, with the number of chains set to four. The value of *R*-hat for all parameters was equal to 1.0, indicating convergence across the four chains (Stan Development Team, 2020).

Finally, to seek a significant relation with individual trait differences measured by questionnaires, the correlation coefficient was calculated using individual parameters (i.e., $u_{n}$ ) of the final model. All analyses were performed using software (*R* statistical package, ver. 4.0.3) along with the “cmdstanr,” “psych,” “rstan,” and “tidyverse” packages (Wickham et al., 2019; Gabry and Češnovar, 2020; Stan Development Team, 2020; Revelle, 2021).

## Results

### Model Comparisons

First, to ascertain the best-fitted model, we computed Widely Applicable Information Criteria (WAIC; Watanabe, 2010) for Model 1, Model 2, and Model 3.

Using WAIC, we found that Model 3 (WAIC = 105685.61, SE = 0.41) was superior to Model 1 (WAIC = 109070.36, SE = 0.23) and Model 2 (WAIC = 110615.55, SE = 0.27). These findings indicated that the processes of perceiving, sharing, and eliciting emotional states differ for anger and happiness, which is consistent with hypotheses 1 and 2.

**Figure 3:**
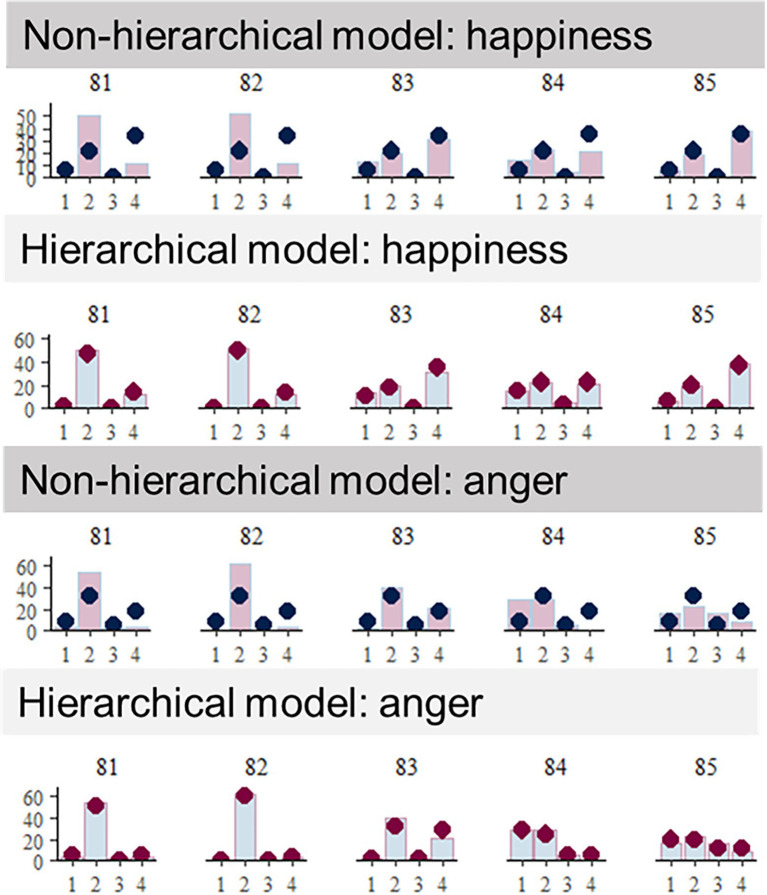
Individual data of Model 3 (non-hierarchical model) and of Model 4 (hierarchical model). For illustration, data of the five participants were selected. The bar signifies observed behavior; the dot denotes the predictive posterior value by the model. Sufficient overlaps between the bars and the dots indicate that the individual observed behaviors were well predicted by the (hierarchical or non-hierarchical) model.

Next, we compared Model 3 and the hierarchical version of that (Model 4). Model 4 was also superior in WAIC (WAIC = 102998.77). Furthermore, we examined posterior predictive performance to verify that the predicted value fitted the performance of individual participants well. Figure 3 shows the parts of individual data in the non-hierarchical MPT (Model 3) and the hierarchical MPT (Model 4). As presented visually, the hierarchical model predicted each performance better than non-hierarchical model. Taking an example of participant #82, the non-hierarchical MPT does not fit the predicted value to the data, although the predicted value is sufficiently fitted to the data in the hierarchical MPT. This result indicates that data had a hierarchical structure allowing individual variations among participants, which is consistent with hypothesis 3. Supplemental figures show all individual data comparing predictions obtained using Model 3 with those obtained using Model 4.

To elucidate how the correlation structure between the parameters affected the predictive value and data fitting, we compared Model 4 to Model 5 using WAIC. Results show that Model 5 (WAIC = 98921.59, SE = 33.55) outperformed Model 4 (expected log pointwise predictive density differences for WAIC = −2038.59). This finding supported hypothesis 4, indicating that data had a nested structure for each participant.

The underlying parameters determined using the final Model (Model 5) are presented in Tables 3, 4. Table 3 shows that a happy expression was likely to be judged to have a feeling state more often than when judging feeling states from no facial movement (i.e., random bias). By contrast, an expression of anger is explainable almost entirely, merely by random bias: the experience judgment bias peculiar to an angry expression was apparently small. Another major difference that could engender insights was that the elicitation parameter and the sharing parameter were equally high for happy facial expressions, whereas only the elicitation parameter was high for angry facial expressions and the sharing parameter was very small. The findings indicated that, although both emotional elicitation and sharing emotion were observed for happy facial expressions, only emotional elicitation was functioning, rather than sharing emotion for angry facial expressions.

**Table 3:** All parameters in Model 5.

	MAP	2.5%	97.5%
Judgment_Ha	0.382	0.323	0.433
Elicitation_Ha	0.865	0.804	0.911
Sharing_Ha	0.965	0.921	0.987
Judgment_An	0.070	0.035	0.120
Elicitation_An	0.840	0.779	0.888
Sharing_An	0.006	0.001	0.027
Response Bias	0.164	0.120	0.214

Subscripts Ha and An, respectively, denote happiness and anger. MAP denotes maximum a posterior.

**Table 4:** Correlation among all parameters.

	Judgment_Ha	Elicitation_Ha	Sharing_Ha	Judgment_An	Elicitation_An	Sharing_An
Judgment_Ha						
Elicitation_Ha	−0.06 [−0.28, 0.18]					
Sharing_Ha	−0.10 [−0.43, 0.25]	−0.24 [−0.55 0.13]				
Judgment_An	**0.54 [0.27, 0.71]**	0.00 [−0.25 0.23]	−0.11 [−0.44 0.25]			
Elicitation_An	−0.12 [−0.34 0.12]	0.21 [−0.01 0.41]	0.30 [−0.01 0.61]	**−0.27 [−0.48 –0.01]**		
Sharing_An	**−0.46 [−0.73–0.03]**	0.20 [−0.19 0.52]	−0.34 [−0.68 0.08]	−0.22 [−0.64 0.20]	−0.05 [−0.42 0.29]	
Response Bias	**−0.28 [−0.47–0.04]**	−0.18 [−0.40 0.05]	0.00 [−0.32 0.34]	**−0.51 [−0.68–0.28]**	0.36 [−0.05 0.63]	−0.10 [−0.29 0.14]

Maximum a posterior (MAP) [95% credible intervals]. Bold typeface signifies that the 95% credible interval of the parameters does not include zero.

As shown in Table 4, the judgment of having an experience was confirmed as similar to those of anger and happiness (*r*[95%CI] = 0.54[0.27, 0.71]). These were correlated negatively with the tendency to judge a feeling state from an expression that has no facial movement (anger, *r*[95%CI] = −0.51[−0.68, −0.28]; happy, *r*[95%CI] = −0.28[−0.47, −0.04]).

### Individual Differences

This section presents exploration of Bayesian Pearson correlations among the individual differences measured using questionnaires and underlying parameters for each participant using JASP (JASP Team, 2021). No scale was found to be correlated with the estimated parameters (all BF_10_ < 2.13: Table 5).

**Table 5:** Bayesian Pearson correlations between the underlying parameters from the MPT model and the rated subscale using questionnaire.

Bayesian Pearson correlations variable	ECS	EC	FS	PT	PD	SPS	SIAS
Judgment_HaPearson’s r	0.00	−0.05	−0.10	0.09	−0.13	−0.02	0.12
BF₁₀	0.13	0.15	0.20	0.19	0.28	0.14	0.25
Upper 95% CI	0.20	0.16	0.11	0.29	0.08	0.19	0.09
Lower 95% CI	−0.21	−0.25	−0.30	−0.12	−0.33	−0.23	−0.32
Sharing_HaPearson’s r	−0.13	0.02	−0.05	−0.10	0.13	0.03	0.06
BF₁₀	0.27	0.14	0.15	0.20	0.27	0.14	0.16
Upper 95% CI	0.08	0.23	0.16	0.11	0.32	0.23	0.26
Lower 95% CI	−0.32	−0.18	−0.25	−0.29	−0.08	−0.18	−0.14
Elicitation_HaPearson’s r	0.17	0.11	0.17	0.06	0.03	−0.11	−0.18
BF₁₀	0.47	0.21	0.49	0.15	0.14	0.21	0.58
Upper 95% CI	0.36	0.30	0.36	0.26	0.23	0.10	0.03
Lower 95% CI	−0.04	−0.10	−0.04	−0.15	−0.18	−0.30	−0.37
Judgment_AnPearson’s r	0.01	−0.03	0.00	0.04	−0.12	0.00	−0.08
BF₁₀	0.13	0.14	0.13	0.14	0.24	0.13	0.18
Upper 95% CI	0.21	0.18	0.21	0.25	0.09	0.20	0.13
Lower 95% CI	−0.20	−0.23	−0.20	−0.16	−0.31	−0.21	−0.28
Sharing_AnPearson’s r	0.10	0.08	0.11	−0.02	0.05	0.04	0.02
BF₁₀	0.20	0.18	0.23	0.14	0.15	0.14	0.13
Upper 95% CI	0.30	0.28	0.31	0.19	0.25	0.24	0.22
Lower 95% CI	−0.11	−0.13	−0.10	−0.22	−0.16	−0.17	−0.19
Elicitation_AnPearson’s r	0.13	0.19	0.09	0.04	0.25	−0.02	−0.03
BF₁₀	0.26	0.61	0.19	0.14	2.13	0.13	0.14
Upper 95% CI	0.32	0.38	0.29	0.24	0.43	0.19	0.18
Lower 95% CI	−0.08	−0.02	−0.12	−0.17	0.04	−0.22	−0.23
Response Bias Pearson’s r	0.05	−0.08	−0.03	−0.07	0.00	−0.02	−0.02
BF₁₀	0.15	0.17	0.14	0.16	0.13	0.13	0.13
Upper 95% CI	0.25	0.13	0.18	0.14	0.21	0.19	0.19
Lower 95% CI	−0.16	−0.28	−0.23	−0.27	−0.20	−0.22	−0.22

ECS, emotional contagion scale; EC, empathic concern in IRI; PT, perspective taking in IRI; PD, personal distress in IRI; FS, fantasy in IRI; SPS, social phobia scale; SIAS, social interaction anxiety scale.

## Discussion

With the MPT model, this study untangled sharing and elicitation (not-sharing) processes that underlie emotion sharing. Moreover, this study quantified the effects of authentic information about expressers’ feeling states perceived from facial expressions. Then, this study was conducted to evaluate the five hypotheses presented below:

1. Angry and happy expressions have distinct inferential processes for perceiving authentic information of feeling states.
2. Processes of sharing and eliciting emotional states differ for anger and happiness.
3. Data have a hierarchical structure allowing variation among individual perceivers.
4. Data have a correlated structure for each estimated parameter fitting multivariate priors.
5. Patterns of correlation exist between parameters computed using the MPT model and the personality traits.

With a new MPT model that can explain the process to share emotional states, we were able to verify that the results supported hypotheses 1–4. Considering the posterior parameters, we obtained three key findings. First, the ability to perceive authentic information from facial expressions was the highest for happiness followed by no facial movement (i.e., random bias); the lowest was anger. Second, whereas both emotional elicitation and sharing emotion were observed for happy facial expressions, only emotional elicitation was visible rather than sharing emotion for angry facial expressions. Third, the parameter used to detect an anger experience was similar to that for happiness; these were negatively correlated with the tendency to perceive authentic information from the expression that has no facial movement. Furthermore, almost no correlation was found between parameters extracted from this experimental task and the social-cognition-related questionnaire (i.e., emotional contagion, empathy, and social anxiety). The results were inconsistent with findings obtained from earlier studies that have investigated the ability to detect emotion and emotional contagion (e.g., Manera et al., 2013; Calvo et al., 2018; Neves et al., 2018; Dawel et al., 2019).

The processes of evoking an emotional state from perceptions of facial expressions are apparently different for anger and happiness. Although this difference has long been argued and explored through several studies (e.g., Deng and Hu, 2018; Murphy et al., 2018), the current study provided the first empirical evidence indicating that the process can quantitatively differ in terms of the ability to detect authentic information. Particularly, a happy expression is easily perceived as authentic information: the perceiver can readily share and induce positive feeling states from that expression. Positive emotions are well known as tending to be shared more than negative emotions (Hinsz and Tomhave, 1991; Hess and Fischer, 2013). Manera et al. (2013) also demonstrated that susceptibility to emotional contagion for positive emotions engenders categorization of most of the faked smiles as authentic. This behavioral tendency can be regarded as a false alarm. However, if so, no relation between the parameter to perceive authentic information of a feeling state from a happy expression and emotional contagion scale by which the current study found was by no means consistent. Interpretation of this discrepancy is difficult, but it might be attributable to the questionnaire which was used. Manera et al. (2013) used the emotional contagion scale by dividing it into positive and negative emotions, but the present study did not apply that distinction; nor have some other studies (Neves et al., 2018; Lima et al., 2021). As with the MPT parameters in the current study, it is desirable to use questionnaires according to differences in emotional valence. More specifically, susceptibility to emotional contagion for positive emotions is expected to lead to the ability to perceive authentic information from happy expressions, whereas that for negative emotions leads to the ability to perceive authentic information from anger expressions.

As for angry facial expressions, the ability to perceive authentic information has a strong negative correlation with the ability to perceive that from no facial movement. The factor that causes the feeling states of perceivers was not a sharing parameter but rather an elicitation parameter. In other words, the ability to perceive authentic information from anger expression is explainable mostly by the random bias to neutral expressions. Although the perceiver can feel a negative state from anger expressions, they are rarely shared. This result indicates that the specific system for over-detecting the emotional experience of anger is apparently small. In the field of emotional mimicry, several findings have supported that anger has not been shared in many situations (Häfner and IJzerman, 2011, Study 2; Hess and Fischer, 2014). Our data further demonstrate that elicited negative experiences from anger expressions should not be regarded as “sharing emotional states” quantitatively. Consequently, when investigating the process of emotional contagion, one must examine differences in valence specifically. Moreover, facial expressions can convey social signals as well as emotional information. Showing happiness typically transmits affiliative intentions, whereas showing anger signals dominance (Hess et al., 2000). For non-affiliative partners through facial expressions, it is highly likely that the sharing process will not occur. In other words, it is possible that another aspect, including the social messages caused by the perception of facial expressions, cuts the sharing process.

Unexpectedly, no robust correlation structures were found between the parameter to perceive the target feeling states from emotional expressions and the parameter to share and elicit the corresponding feeling states. Multiple points of theoretical and empirical evidence have been accumulated based on assumed connections between the perception of emotion and the sharing/arousal emotion (Hatfield et al., 2009; Sato et al., 2013; Wood et al., 2016). Nevertheless, no correlation was found between these parameters in this study: the tendency to perceive the authentic information from emotional expressions does not cause an ability to share or arouse from emotional expressions. Similarly, comparison of the models revealed that a hierarchical model with a correlation structure among these parameters was preferable to a model without such a structure from the perspective of WAIC. These two facts suggest that each process interacts organically as a system, but no strong correlation exists. For the current study, we provided mathematical expressions of the respective parameters. The data and codes used for them are also disclosed in OSF,^4^ which is a bridge to future research. Ascertaining which parameters physically correspond to which neural area is of particular interest. Tackling such questions can be expected to provide deeper insights into the system used for perceiving information from facial expressions of emotion.

Regarding correlation between parameters computed using the MPT model and traits measured by the questionnaires, only slight correlation was found. This result indicates that parameters estimated using the current MPT model must be interpreted carefully because of the lack of external validity using questionnaires (Bott et al., 2020). The results might be observed because of fact that all the facial expressions used for the current study were posed expressions instead of genuine displays. The participants’ responses to detect an experience from perceived facial expressions can be regarded as a false alarm, which is conceptually different from the ability to discern authentic information from genuine expressions (Namba et al., 2018; Zloteanu et al., 2018). The current study specifically addressed “how the authentic information actually received by perceivers influences the process of sharing emotional states.” The process to sharing emotional states is essentially a phenomenon on the perceiver side rather than on the expresser side. Therefore, emphasis on the interpretation of perceiver can be justified in terms of information theory (Shannon, 2001; Jack et al., 2014) and affective pragmatics (Scarantino, 2017). The other concern is what is measured by the questionnaire. Indeed, several researchers who investigated relations between trait-relevant self-reports and actual performance derived from the psychological experiment reported that participants might lack insight (or metaknowledge) into their own relative level of skill (Ickes, 1993; Dunning et al., 2003). Using an analysis with response style (e.g., Bolt et al., 2014) might be useful to delve into the concepts reflected in the questionnaire.

Although we were able to develop a new computational model of the process to share emotional states, and although we provided the first evidence for the related issues, it is noteworthy that there were some limitations to this study. In this study, as an antecedent of sharing emotional states, the judgment of authentic information by the perceiver was conditioned in the MPT model (Figure 1). This order is aligned with the experimental flow which the current study used, but it is not clearly supported by evidence. Based on Simulation of Smiles Model (SIMS; Niedenthal et al., 2010), facial mimicry and its feedback theoretically lead to the interpretation of facial expressions including judgment of authenticity (Maringer et al., 2011; Korb et al., 2014). McGettigan et al. (2015) also reported sensorimotor systems as related to authenticity judgments. Consequently, that avenue of research has been regarded as the opposite direction for the process that the current study targeted. Future studies must be undertaken, as Sato et al. (2017) did, to examine the dynamic pattern in the brain during perceiving facial expressions to clarify which order is the fixed or varied pattern. In addition, the authenticity judgments should be made with a rating scale because yes-or-no response provides much less information than a rating scale about the relative perceived genuineness of different stimuli (Dawel et al., 2017). Next, mimicry has been treated as an important phenomenon both for deciphering information *via* facial expressions and in the emergence of emotional experiences from facial expressions, including emotional contagion. However, this index was not used for this study. For that reason, the current study incorporated only the forward process and the empathetic process into the model, but not the imitation process (Elfenbein, 2014). If facial mimicry was included in the dependent variables, a further MPT model that can explain emotion sharing, including imitation process, can be verified empirically. To delve into the order of each process in a sharing emotion model, the imitation process and the judgment process in the MPT can be interchanged and examined. Adding facial mimicry, which is a fundamentally important variable for emotional contagion, to the current MPT model will improved as a more comprehensive model of emotional contagion: this research was positioned to provide budding findings for leveraging such studies.

In sum, we formally modeled the process to share emotional states from facial expressions. By building computational models, we advance the scientific study of sharing emotion. First, the ability to perceive authentic information of feeling states from a happy expression was a higher probability than the probability of judging authentic information from an anger expression. Next, happy facial expressions can activate both emotional elicitation and shared emotions in perceivers, whereas only emotional elicitation functions, rather than sharing emotion, for angry facial expressions. Third, the parameter to detect having an anger experience was found to be positively correlated with that of happiness. Finally, only weak correlation was found between the parameters extracted from this experimental task and the questionnaire measured emotional contagion, empathy, and social anxiety. The current study, which disclosed codes that can replicate the same MPT models, provides a new computational approach to evaluation of the perception of facial expression and emotional contagion and to advance affective science research.

## Data Availability Statement

The datasets presented in this study can be found in online repositories. The names of the repository/repositories and accession number(s) can be found at: https://osf.io/hyv2k/.

## Author Contributions

NS conceived and conducted the experiment, performed statistical analysis, and figure generation. WS, KN, and KW discussed the results and contributed to the final manuscript. All authors reviewed the manuscript. All authors contributed to the article and approved the submitted version.

## Funding

This research was supported by Early-Career Scientists (20K14256) from JSPS to SN, Early-Career Scientists (19K20387) from JSPS to KN, Grant-in-Aid for Scientific Research on Innovative Area (17H06344) from JSPS, and by Moonshot R&D (JPMJMS2012) from JST to KW.

## Conflict of Interest

The authors declare that the research was conducted in the absence of any commercial or financial relationships that could be construed as a potential conflict of interest.

## Publisher’s Note

All claims expressed in this article are solely those of the authors and do not necessarily represent those of their affiliated organizations, or those of the publisher, the editors and the reviewers. Any product that may be evaluated in this article, or claim that may be made by its manufacturer, is not guaranteed or endorsed by the publisher.

## Footnotes

^1^ www.crowdworks.jp

^2^ www.gorilla.sc

^3^ https://osf.io/hyv2k/?view_only=71b1c20841cc4c0687fa17a3d6114c6a

^4^ https://osf.io/hyv2k/?view_only=71b1c20841cc4c0687fa17a3d6114c6a
