# Supplementary material for: Computational Process of Sharing Emotion: An Authentic Information Perspective
Source: Front Psychol. 2022 May 12;13:849499. doi: 10.3389/fpsyg.2022.849499 (PMC9134197; doi:10.3389/fpsyg.2022.849499)
Supplement: Supplementary file 1 [file Data_Sheet_1.docx]

Supplementary Material

# Supplementary Tables

Table 1. Comparison of questionnaire responses with those of earlier studies of translated versions: ECS, Emotional Contagion Scale; SPS, Social Phobia Scale; SIAS, Social Interaction Anxiety Scale

|  |  |  |  |  |  |
| --- | --- | --- | --- | --- | --- |
| Subscale | *Earlier studies* | μ | *t* | *p* | *g* [95%CI] |
| ECS | Kimura et al. (2007) | 2.88 | 4.44 | 0.00 | 4.43 [ 2.37, 6.49] |
| Empathic Concern (IRI) | Himichi et al. (2017) | 3.31 | 2.13 | 0.04 | 2.12 [ 0.15, 4.10] |
| Perspective Taking (IRI) | Himichi et al. (2017) | 3.02 | 0.57 | 0.57 | 0.57 [-1.38, 2.53] |
| Personal Distress (IRI) | Himichi et al. (2017) | 3.17 | 0.33 | 0.75 | 0.32 [-1.63, 2.28] |
| Fantasy (IRI) | Himichi et al. (2017) | 3.16 | 0.31 | 0.76 | 0.31 [-1.64, 2.27] |
| SPS | Kanai et al. (2004) | 0.95 | 1.17 | 0.25 | 1.16 [-0.80, 3.12] |
| SIAS | Kanai et al. (2004) | 1.50 | 0.80 | 0.42 | 0.80 [-1.16, 2.76] |
|  |  |  |  |  |  |

Table 2. Correlation matrix for questionnaires: ECS, Emotional Contagion Scale; EC, Empathic Concern in IRI; PT, Perspective Taking in IRI; PD, Personal Distress in IRI; FS, Fantasy in IRI; SPS, Social Phobia Scale; SIAS, Social Interaction Anxiety Scale

|  |  |  |  |  |  |  |  |
| --- | --- | --- | --- | --- | --- | --- | --- |
| Subscale | ECS | EC | PT | PD | FS | SPS | SIAS |
| ECS | 1 |  |  |  |  |  |  |
| Empathic Concern (IRI) | 0.05 | 1 |  |  |  |  |  |
| Perspective Taking (IRI) | -0.04 | 0.69 | 1 |  |  |  |  |
| Personal Distress (IRI) | 0.04 | 0.09 | -0.04 | 1 |  |  |  |
| Fantasy (IRI) | -0.01 | 0.46 | 0.45 | 0.30 | 1 |  |  |
| SPS | 0.01 | 0.05 | 0.03 | 0.11 | -0.05 | 1 |  |
| SIAS | -0.05 | -0.02 | -0.13 | -0.13 | -0.13 | 0.18 | 1 |
|  |  |  |  |  |  |  |  |


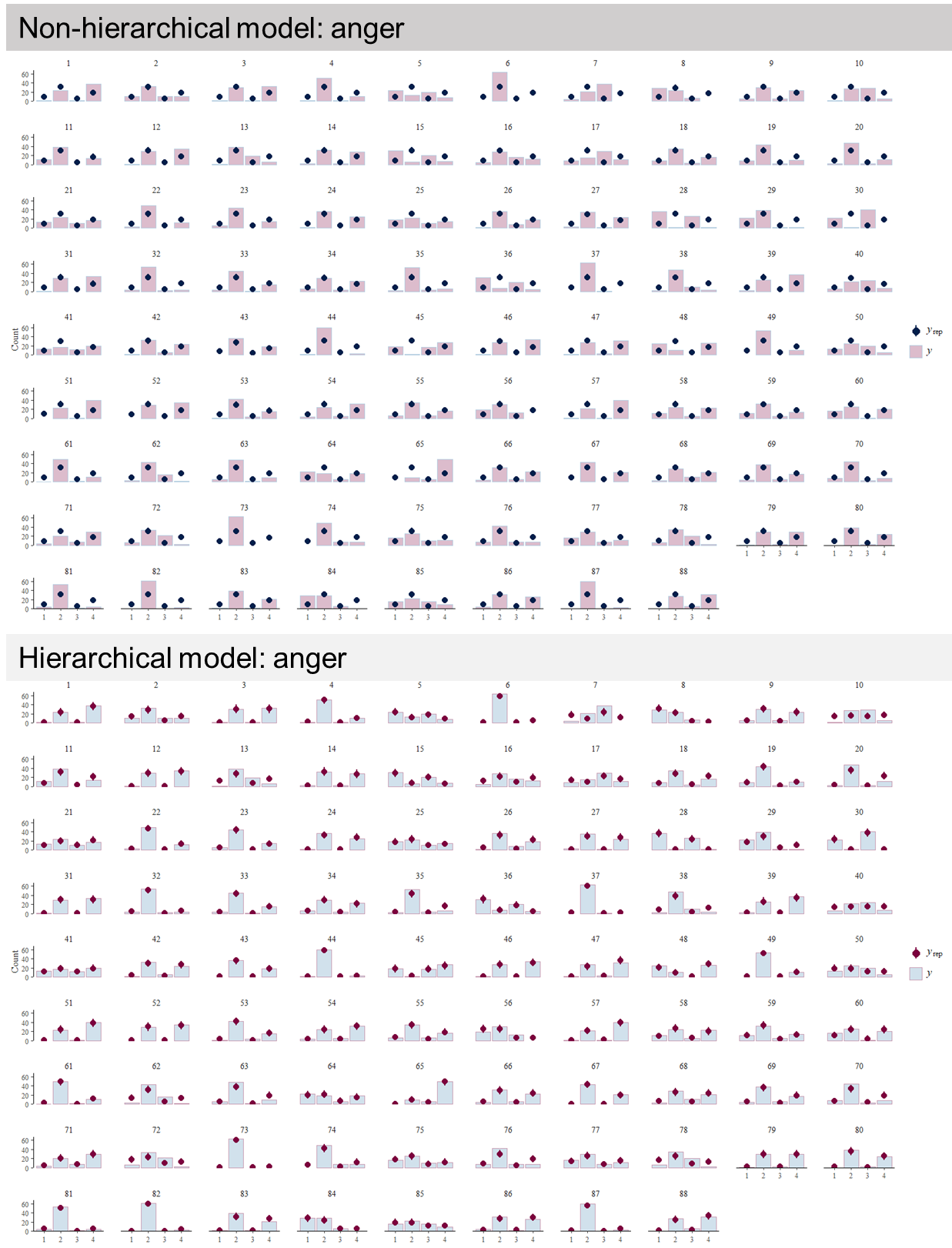


Supplemental Figure 1. Individual data of Model 3 (non-hierarchical model) and of Model 4 (hierarchical model) for anger. The bar signifies observed behavior; the dot denotes the predictive posterior value by the model.


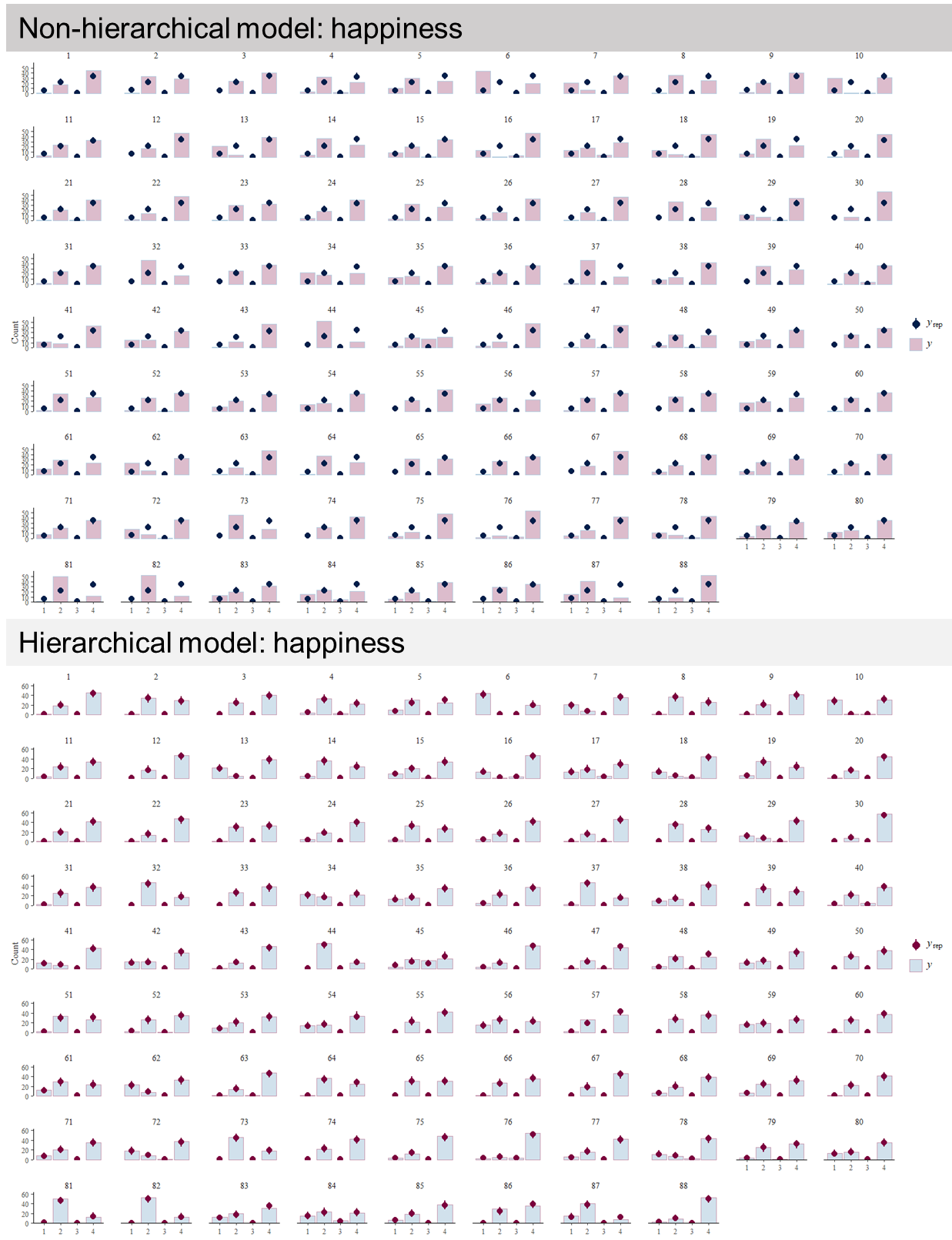


Supplemental Figure 2. Individual data of Model 3 (non-hierarchical model) and of Model 4 (hierarchical model) for happiness. The bar signifies observed behavior; the dot denotes the predictive posterior value by the model.
